# Potential targets for molecular imaging of apoptosis resistance in hepatocellular carcinoma

**DOI:** 10.2349/biij.7.1.e5

**Published:** 2011-01-01

**Authors:** Z Liu, M Cheng, MZ Cao

**Affiliations:** Department of Nuclear Medicine, Third Affiliated Hospital of Sun Yat-Sen University, Guangzhou, China

**Keywords:** Molecular imaging, apoptosis, hepatocellular carcinoma

## Abstract

Hepatocellular carcinoma (HCC) is one of the most common cancers, which is mainly a concern in Southeast Asia. Apoptosis resistance in HCC is one of the significant factors for hepatocarcinogenesis and tumour progression. Recent advances of apoptosis resistance mechanisms in HCC could serve as potential targets for molecular imaging, which would be of considerable value to explore the molecular processes involved in HCC progression and to evaluate responses of certain anti-HCC therapies. Disruptions in the balance of anti-apoptotic and pro-apoptotic processes have been found to be involved in apoptosis resistance in HCC. Loss of response to death receptors, transformation of growth factor-β induced apoptosis, upregulation of anti-apoptotic Bcl-2 subgroup, as well as downregulation of pro-apoptotic Bax subgroup and BH3-only subgroup, are associated with apoptosis resistance in HCC. Mutation of p53 gene, dysregulation of NF-κB and survivin are also of interest because of their contribution to HCC development. In this review, the aim is to identify potential targets for molecular imaging of apoptosis resistance in HCC.

## INTRODUCTION

Molecular imaging, which uses tracers that bind specifically to molecular targets [1], is an emerging field that aims to integrate patient-specific and disease-specific molecular information with traditional anatomical imaging readouts [2]. Molecular imaging is not only based on nuclear medicine, but also on magnetic resonance imaging (MRI), computed tomography (CT), and the emerging field of optical imaging. It provides visualisation in space of normal as well as abnormal cellular processes at the molecular or genetic level, rather than at the anatomic level [3]. It has its roots both in molecular biology and imaging technologies. On one hand, progress in our understanding of the molecular biology of diseases will accelerate the development of molecular imaging and lead to more effective therapeutic strategies. On the other hand, development of different non-invasive imaging technologies provide the technological foundation for molecular imaging.

Apoptosis, also known as programmed cell death, is an indispensable component of normal human growth and development, immunoregulation and homeostasis. It was characterised by morphological features such as cytoplasmic shrinkage, chromatin condensation in the nucleus, phosphatidylserine (PS) exposure, plasma membrane blebbing and disintegration of the cell into small fragments (apoptotic bodies) that are engulfed by nearby cells [4]. Apoptosis is nature’s primary component of cell proliferation and growth. It represents a physiological way to eliminate excess cells during liver cell development and regeneration [5]. Resistance to apoptosis is associated with carcinogenesis and progression, during which cells often show alteration in the genes regulating apoptosis machinery. Apoptosis can also augment the escape of tumour cells from surveillance by the immune system [6, 7].

Hepatocellular carcinoma (HCC) is one of the most common cancers worldwide with the highest prevalence in Southeast Asia [8]. The apoptosis resistance of hepatic cells was reported to be one of the significant factors for hepatocarcinogenesis or tumour progression in HCC [9]. However, the apoptosis resistance in HCC is complicated. It is necessary to investigate the molecular mechanisms responsible for apoptosis resistance contributing to HCC development. These molecules can be further used as potential targets for molecular imaging to visualise the molecular processes in HCC development as well as to evaluate the responses of certain anti-HCC therapies. This review is an effort to update the recent relevant contributions that report molecular targets involved in apoptosis resistance in HCC.

## MOLECULAR BIOLOGY

The molecular biology of apoptosis resistance in HCC may offer a road map for the development of molecular imaging strategies. They determine the regulatory genes and proteins involved in apoptosis resistance in HCC that may provide potential targets for molecular imaging.

### Component of apoptosis pathway

**Figure 1:**
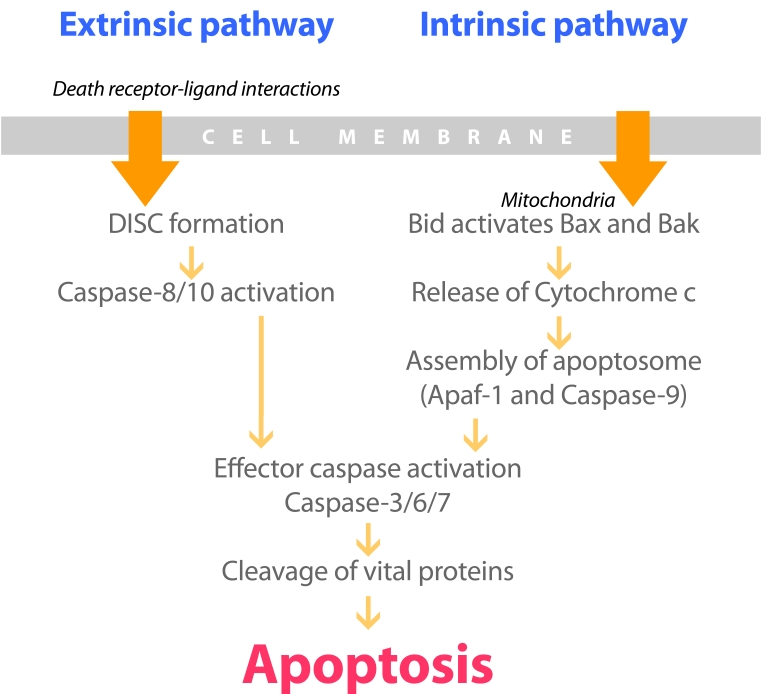
Component of apoptosis pathways

There are two distinct pathways of signal transduction of apoptosis that exist in mammalian cells (Figure 1) [10, 11]. One is the extrinsic cell death pathway, which is characterised by the activation of cell surface death receptors following binding of their specific ligands. It is mediated by death receptors, a subgroup of the tumour necrosis factor (TNF) receptor superfamily, including CD95 (also known as Fas), TNF-related apoptosis-inducing ligand (TRAIL), and TNF-R1. All death receptors are characterised by an intracellular motif called death domain (DD). Death receptor-mediated cell death is initiated by the recruitment of adapter proteins, like Fas-associated death domain (FADD), via DD, which then bind to the death domain-containing caspase-8 or -10, to form the death-inducing signaling complex (DISC). DISC formation results in the activation of caspase-8, which then directly cleaves and activates caspase-3, -6, or -7, the executioner enzymes of apoptosis.

The other is the intrinsic or mitochondrial pathway, which is initiated by multiple forms of cellular stress, including radiation and chemotherapeutic drugs. In this pathway, pro-apoptotic Bcl-2 family members Bax and Bak translocate to mitochondria. The Bcl-2 homology domain 3 only (BH3-only) protein Bid activates Bax and Bak to mediate the release of cytochrome c into cytosol. This triggers the assembly of the apoptosome (Apaf-1 and caspase-9) and, subsequently, the activation of caspase-3, -6, or -7, and cell death. The X-linked inhibitor of apoptosis (XIAP) may bind directly to caspases and inhibit their enzymatic activity. The inhibitory function of XIAP is countered by the second mitochondrial-derived activator of caspase (Smac) and Omi/HtrA2, which are also released into cytoplasm.

These two principle pathways may proceed completely independently in many cell types, but there is also considerable cross-talk between extrinsic and intrinsic pathways [12]. A mechanism by which the mitochondrial pathway is engaged, following death receptor activation, is through caspase-8-mediated cleavage of Bid, a pro-apoptotic member of the Bcl-2 family. Once cleaved, truncated Bid (tBid) translocates to mitochondria, where it can induce the release of cytochrome c, SMAC and Omi/HtrA2. In this manner, death receptor signals may be amplified through formation and activation of the apoptosome, which can increase the activation of effector caspases.

### Regulation of death-receptor pathway

Tumourigenic disruption in death-receptor pathway or extrinsic pathway occurs less frequently than in mitochondria pathway or intrinsic pathway. Nevertheless, tumour cells are often resistant to death-receptor-mediated apoptosis. Furthermore, mutations in CD95 (Fas), TRAIL receptor and downstream signaling pathway do occur in cancer [13].

1. Fas: The majority of HCC show one or more alterations in the Fas molecules, which inhibit Fas-mediated apoptosis [14]. Loss of response to Fas in HCC may be produced either by downregulation of Fas expression [14, 15], concomitant with decreased expression of downstream molecules, such as FADD or caspase-8 (also known as FLICE) [15], or by upregulation or overactivation of molecules that counteract its pro-apoptotic effect, including nuclear factor-kappa B (NF-κB), Bcl-2 or Bcl-X_L_ [16–18]. The status of Fas and Fas ligand (FasL) expression can also predict hepatocellular carcinoma recurrence [19].
2. TRAIL: TRAIL and specific agonistic antibodies have attracted considerable attention for their potential use in selective induction of apoptosis in activated stellate cells, as well as in virus-infected and malignant hepatocytes without associated collateral damage to normal hepatocytes [20]. Loss of TRAIL-R may promote growth of neoplastic changes in liver, and this may possibly be a result of blocked apoptosis in HCC [21]. HCC constitutively express TRAIL mRNA and proteins, but most HCC are insensitive towards TRAIL-mediated apoptosis. Hepatitis B virus core protein [22] and overactivation of NF-κB and Bcl-X_L_ [23] in HCC have been indicated to restrain the TRAIL-induced apoptosis. Efficacy of TRAIL-induced apoptosis in tumours *in vivo* has been reported to be monitored by dual enzyme substrate (Rluc/Fluc) imaging, which demonstrated its role in inducing apoptosis in neoplastic cells [24].
3. cFLIP: Cellular FLICE/caspase-8 inhibitory protein (cFLIP), which is an intracellular inhibitor of caspase-8 activation that potently inhibits death receptor-mediated apoptosis, was found to be constitutively expressed in all human HCC cell lines, and was expressed more in human HCC tissues than in non-tumour liver tissues [25].
4. BRE: Brain and reproductive organ-expressed protein (BRE), as a death receptor associated protein, specifically down-modulates death receptor-mediated apoptosis by inhibiting activation of the mitochondrial apoptotic pathway. It was found to bind the death receptors TNF-R1 and Fas, and upon over-expression, conferred resistance to apoptosis induced by TNF-α, anti-Fas agonist antibody, cycloheximide, and a variety of stress-related stimuli [26]. Thus, BRE is considered anti-apoptotic *in vivo* and may promote tumourigenesis when overexpressed, with marked over-expression of BRE detected in the majority of HCC [27].

### Regulation of mitochondrial pathway

The Bcl-2 proteins are the crucial checkpoints for the intrinsic or mitochondrial pathways [28]. Bcl-2 family proteins are structurally defined by their Bcl-2 homology domain (BH domains) into multidomains and BH3-only, and are functionally categorised into anti-apoptotic and pro-apoptotic [29]. Bcl-2 family members are generally categorised into three subgroups. The first group includes Bcl-2, Bcl-X_L_, Mcl-1, Bcl-w, Bcl-B/Nrh/NR13 and Bfl-1/Bcl-2A1/GRS. These molecules contain multi-BH domains and function to inhibit apoptosis. The second group also contains multi-BH domains but the proteins function to promote apoptosis. These proteins include Bax, Bak, Bok/Mtd and Bcl-X_S_. The third group contains only a BH3 domain and it includes Bid, BAD, Bik/Nbk, Bim/Bod, PUMA, Noxa, Hrk/DP5, Bmf, Spike and Bnip proteins. The BH3-only proteins bind and regulate the pro-survival Bcl-2 family members to promote apoptosis.

Many of the genetic alterations observed in HCC lead to an imbalance in pro-apoptotic and anti-apoptotic members of the Bcl-2 family [30].

1. Bcl-2 subgroup: Bcl-XL and Mcl-1 are over-expressed in a great percentage of HCC cells, including HepG2, Hep3B, Huh7 cells and human HCC tissues. The expression of Mcl-1 is correlated with Bcl-XL in HCC tissues [31, 32]. Bcl-XL is also a significant prognostic factor for disease progression and poorer survival in human HCC [33].
2. Bax subgroup: In contrast, pro-apoptotic members of the family, such as Bax or Bcl-XS are downregulated in HCC with dysfunction in the p53 pathway [33].
3. BH3-only subgroup: Members of the BH3-only family, such as Bid, show decreased expression in HCC related to hepatitis B virus X protein or hepatitis C virus polyprotein [34]. Bid can also block the inhibitory effect of Bcl-2 on Fas-mediated apoptosis of HCC cell line BEL-7404 cells [35].
4. XIAP: XIAP, a well-known inhibitor of caspases, was reported to be overexpressed in HCC, and inversely correlated with apoptosis [36]. Further, studies in established HCC cell lines with different metastatic capabilities indicated a correlation of metastasis with resistance to apoptosis and increased expression of XIAP [37].

Caspases are attractive targets because of their central role in the execution of cell death [38]. Both the death receptor and mitochondrial pathways of apoptosis eventually activate several effector caspases [38]. As caspases play vital roles in mediating the initiation and propagation of the apoptotic cascade, the ability to image caspases activation non-invasively will provide an opportunity to evaluate the regulation of apoptosis status *in vivo*.

Several strategies are emerging to image the activation of caspases. Caspase activities have been imaged using exogenous radio-labeled imaging agents. In a series of cell-based experiments, two radio-iodinated peptides selective for caspase-3 were found to have increased uptake and retention within apoptotic cells [39]. Zhou *et al*. [40] employed an ^18^F-radio-labeled isatin sulfonamide analog that targets activated caspase-3. This agent was used with micro-PET imaging to visualise cyclohexamide-induced hepatic apoptosis in a rat model. One strategy utilised a polymer conjugated to a near-infrared fluorescence effector caspase-specific peptide to create a biocompatible, cell-permeable, autoquenched nanoparticle to image apoptosis in real time in living cells [41]. Another approach successfully employed a novel caspase-1 specific, activatable, near-infrared fluorescent probe as a means to image apoptosis in mice models of apoptosis [42]. Recent reports have demonstrated that caspase-specific fluorescently labeled activity-based probes (ABPs) have the potential to be used for non-invasive imaging of apoptosis in both pre-clinical and clinical settings [43]. Although work on imaging the activation of caspases is promising, there is still limited animal data so far and no human data.

### Regulation of p53

Human tumours harbour mutation, which renders them resistant to apoptosis. The p53 gene is one of the most frequently mutated genes in tumour cells [12]. The tumour suppressor protein p53, a transcription factor, can prevent cells from growth and division, thus mediating cell-cycle arrest, DNA repair and apoptosis, after the p53 gene has been activated by multiple forms of cellular stresses [44]. Abnormalities of p53 are considered a predisposing factor for hepatocarcinogenesis [44].

The p53 gene is frequently mutated in high-grade HCC [45]. Bressac *et al*. [46] studied the p53 gene and protein in seven HCC-derived cell lines, and found that six of them showed p53 abnormalities, suggesting that alterations in p53 may be important events in the transformation of hepatocytes into the malignant phenotype [47]. Inactivation of the p53 gene also plays an important role in the progression of chronic liver damage to HCC by directly or indirectly inducing chromosome instability, cell proliferation and neovascularisation [48]. It was reported that attenuated p53 function and telomere-induced chromosomal instability play a critical and cooperative role in the progression of chronic liver damage to HCC, and the loss of p53 expression or the presence of abnormal forms of the protein is frequently associated with HCC cell lines [49]. Furthermore, it was reported that p53-dependent gene expression has already been imaged *in vivo* with positron emission tomography (PET) and by *in situ* fluorescence [50]. A retroviral vector (Cis-p53/TKeGFP) was generated by placing the herpes simplex virus (HSV) type 1 thymidine kinase (tk) and enhanced green fluorescent protein (GFP) fusion gene under the control of a p53-specific response element. Apoptosis-induced upregulation of p53 transcriptional activity was demonstrated by using PET imaging, which would be of considerable value in assessing novel therapeutic approaches that are mediated through p53-dependent pathways.

### Regulation of NF-κB in inflammation

Recent studies have indicated that virus activity may drive tumour progression [51] and NF-κB is considered to be an important molecular link between chronic inflammation and cancer development [6, 52, 53]. NF-κB was initially discovered as a transcription factor in the nucleus of B cells that binds to the enhancer of the kappa light chain of immunoglobulin, and it has since been shown to be expressed ubiquitously in the cytoplasm of all types of cells [54]. Its family members may accumulate in the cytoplasm and reach the nucleus where they transactivate several growth-related genes and anti-apoptotic gene [55]. NF-κB suppresses apoptosis by inducing the expression of a number of genes whose products inhibit apoptosis, including IAPs, cFLIP, TNF receptor associated factor 1 (TRAF1) and TRAF2. Two typical prosurvival NF-κB targets are Bcl-X_L_, an anti-apoptotic member of Bcl-2 family, and XIAP, a member of the caspases inhibitor, which are frequently overexpressed in HCC [56]. These anti-apoptotic proteins have been found to work in a coordinated fashion to block apoptosis at multiple steps along the apoptotic cascade or to regulate other pro- or anti-apoptotic pathways [57]. Transactivating forms of NF-κB may upregulate the expression of several other genes involved in anti-apoptosis, cell proliferation and invasion, and drug resistance (e.g., Bcl-2, Bcl-X_L_, cyclin D1, c-Myc, IL-6, COX-2, iNOS, MMPs) [37]. Moreover, other hallmarks of cancer such as sustained angiogenesis, tissue invasion, and metastasis were also reported to be at least partially dependent on NF-κB signaling [58, 59].

HCC commonly develops in the background of chronic hepatitis, in which NF-κB activation is often observed [60]. Certain studies have implicated that members of NF-κB family act in both HBV- and HCV-induced neoplastic development of liver [61]. Overproduction of inflammatory cytokines and growth factors during the early stages of hepatocarcinogenesis deregulates the inducible nitric oxide synthase, inhibition κB (IκB) kinase, and NF-κB axis [55]. It has also been suggested that TNF-α has a central role in activating NF-κB and in protecting transformed hepatocytes against apoptosis [62].

### The TGF-β signal pathway

The transforming growth factor-beta (TGF-β) signaling pathway is a key player in metazoan biology, and its misregulation can result in tumour development [63]. Pathological forms of TGF-β signaling promote tumour growth and invasion, evasion of immune surveillance, and cancer cell dissemination and metastasis [63]. TGF-β1 is also a key cytokine in the regulation of hepatic apoptosis, and act via the Smad pathway to initiate gene transcription and activation of caspases [64]. Blocking TGF-β can upregulate E-cadherin, and reduce migration and invasion of HCC [65]. Huang *et al*. [65] reported that the HCC might also overexpress a specific set of microRNAs that would allow the escape from TGF-β-induced apoptosis, which might be a prerequisite for HCC progression [66].

### A novel protein--survivin

Survivin is a novel protein and also an attractive target which is involved in multiple signaling mechanisms controlling tumour maintenance [67]. Survivin is a member of the inhibitor of apoptosis (IAP) family of proteins, of which eight members are known to exist, i.e., XIAP, NAIP, c-IAP1, c-IAP2, livin, ILP2, BRUCE and survivin [68]. IAPs prevent cell death by acting as endogenous suppressors of caspase activity [69]. Multiple *in vitro* and *in vivo* studies have shown that survivin inhibits cell death, especially apoptosis, through both intrinsic and extrinsic mediators of apoptosis, including IL-3 withdrawal, Fas stimulation, TRAIL, overexpression of Bax, p53, as well as caspase-3, -7 and -8, to name a few [70]. Importantly, it is recognised that survivin not only inhibits apoptosis, but also, as a component of the chromosomal passenger complex, favours cancer cell proliferative activity [36]. Survivin is rarely expressed in normal healthy adult tissues, but upregulated in the majority of cancers [70]. It was found to be overexpressed in HCC cell lines and tissues [71], and might play a pivotal role in metastasis of HCC [72]. It may also be positively correlated with a high risk of disease recurrence and poor prognosis [73].

## IMAGING TECHNOLOGIES

There are mainly three imaging technologies used for molecular imaging of apoptosis, namely MRI, nuclear imaging (quantitative autoradiography, gamma camera, single-photon emission computed tomography (SPECT) and PET), and *in vivo* optical imaging [74–81].

Several ligands with specific, high-affinity binding to phosphatidylserine (PS) have been exploited as agents capable of detecting apoptosis. The most investigated ligand is annexin V, which binds with high affinity to PS [82]. Annexin V-functionalised cross-linked iron oxide (CLIO) was designed as a contrast agent for MRI, which was additionally labeled with Cy5.5 to allow co-localisation with optical imaging techniques [83]. Zhao *et al*. [84] demonstrated success by labeling the C2-domain of a PS binding protein, synaptotagmin I, with superparamagnetic iron oxide nanoparticles (SPIOs). This SPIO-labeled protein was shown to bind to apoptotic cells *in vitro* and detected by MRI.

Nuclear imaging approaches have the advantages of high intrinsic sensitivity, unlimited depth penetration, and a broad range of clinically-available and clinically-tested molecular imaging agents [2]. For example, ^99m^Tc-labeled annexin V is the most extensively investigated and used *in vivo* in detecting apoptosis. Numerous ^99m^Tc-annexin V-based radioligands have been developed and clinical trails have already been undertaken [85]. Yagle *et al.* [86] also indicated that ^18^F-annexin V binds specifically to apoptotic tissues in rat model of liver apoptosis, which may be useful in early assessment of the clinical response to cancer therapy. Some other radioligands for PET imaging of apoptosis were proposed, such as ^123^I-annexin V [87], ^11^C-annexin V [88].

Optical techniques for molecular imaging include optical coherence tomography, fluorescence or luminescence imaging, and infrared imaging, which are commonly used for pre-clinical cellular and molecular imaging in animals [89]. Besides radionuclide imaging, biotin-labelled and fluorescent annexin V have been applied in the detection and confirmation of apoptosis. Fluorescent labeled annexin V has been applied to image cardiomyocyte apoptosis in single cardiomyocytes [90]. Annexin V was successfully conjugated with a near-infrared fluorochrome, cyanine-5.5 (Cy5.5), and applied in detecting apoptotic cells *in vivo* [91, 92]. Jiang *et al*. [93] developed protease reporter molecules based on a fluorophore (Cy5) linked to a polycationic cell-permeant peptide, specific protease cleavage site, and polyanionic peptide. FITC-annexin V is currently used widely as a research reagent for apoptosis detection in flow cytometry [92, 93].

## CONCLUSION AND FUTURE PERSPECTIVES

Molecular imaging is a rapidly emerging field in biomedical research to sense biological processes such as gene expression, apoptosis, etc. The recognition that apoptosis resistance acts as a pathogenic mechanism and contributes to tumourigenesis has gained increasing interest in molecular imaging. In the past couple of years, many strategies about apoptosis resistance in HCC have been reported. Overexpression of anti-apoptotic proteins, such as cFLIP, BRE, Bcl-2 subgroup, XIAP, survivin; downregulation of pro-apoptotic proteins, such as Fas, TRAIL, Bax subgroup, BH3-only subgroup; as well as mutation of p53 gene, activation of NF-κB and escape from TGF-β-induced apoptosis, all contribute to apoptosis resistance in HCC cells and tissues. The identification of these genes and proteins that regulate apoptosis resistance in HCC and the potential application of these molecules as imaging targets could add critical insights into inducing sensitivity to apoptotic pathways in HCC, as well as allow the noninvasive evaluation of the efficacy of therapeutic options for HCC in the near future.

Technologies like MRI, nuclear medicine, and optical imaging techniques are making significant contributions to molecular imaging. It will also be important to continuing developing technologies that combine non-invasive anatomical and functional imaging. These sensitive imaging-based assays would provide a spatial and temporal dimension to monitor molecular processes if the molecular probes of the genes and proteins that regulate apoptosis resistance in HCC were labeled with radionuclides or fluorescence protein, and would be of considerable value in the understanding of apoptosis resistance in HCC. However, the study of MRI, nuclear medicine techniques, and optical imaging techniques to detect and track apoptosis *in vivo* has only just begun. Further work is needed to prove that those techniques would be effective in the detection of apoptosis resistance in HCC, and their clinical applicability still remains to be defined.
